# Right ventricular dysfunction following continuous flow left ventriccular assist device placement in 51 patients: predicators and outcomes

**DOI:** 10.1186/1749-8090-7-60

**Published:** 2012-06-27

**Authors:** Siyamek Neragi-Miandoab, Daniel Goldstein, Ricardo Bello, Robert Michler, David D’Alessandro

**Affiliations:** 1 Department of Cardiothoracic Surgery, Montefiore Medical Center, Albert Einstein College of Medicine, 3400 Bainbridge Ave, MAP Building, 5th floor, New York, NY, 10467, USA

**Keywords:** right heart failure, congestive heart failure, septal function, tricuspid valve, regurgitation

## Abstract

**Background:**

Right ventricular (RV) dysfunction following implantation of a left ventricular assist device (LVAD) is a serious condition and is associated with increased mortality.

**Methods:**

The aim of the study is to investigate the significance of pre-existing RV dysfunction, tricuspid valve (TV) insufficiency, and the severity of septal deviation following LVAD implantation on RV dysfunction, as well as the outcome and short-term complications in 51 patients from June 2006 to August 2010. Student *t* test was used to compare the data and estimate the p value.

**Results:**

Mean age was 55.1 ± 13, with a male to female ratio of 3.25. The 30-day mortality was 13.7% (7/51 patients), and the overall mortality was 23.5% (12/51 patients). Meanwhile, 21 patients (21/51; 41.2%) have undergone orthotopic heart transplantation. The mean time of support was 314.5±235 days with a median of 240 days at the time of closing this study. Echocardiographic evaluation of RV function pre- and post-implantation of an LVAD demonstrated septal deviation towards the left ventricle in immediate postoperative phase, which correlated with acute RV dysfunction (*p* = 0.002). Preoperative RV dysfunction was a significant predictor of postoperative right heart dysfunction following implantation of an LVAD (*p* = 0.001).

**Conclusion:**

Preoperative RV dysfunction is a predictor of RV failure in LVAD patients. The adjustment of septal deviation through gradual increase of the LVAD flow can prevent the acute RV dysfunction following LVAD placement.

## Background

A preoperative RV dysfunction is associated with progressive RV dysfunction and failure following LVAD implantation in patients with congestive heart failure (CHF) [1]. Acute RV dysfunction is a frequent complication of LVAD implantation and has a high rate of morbidity and mortality [2] occurring in greater than 20% of cases [3]. Echocardiography is a useful diagnostic tool to monitor the RV behavior during LVAD support [4]. A change in RV shape and dimension may contribute to RV dysfunction, while this change in RV geometry is a result of disturbed interaction between left ventricle (LV) and RV during LVAD support [5]. A rapid off loading of LV results in shifting of the septum from right to left. This septal deviation towards the LV reduces the contribution of the septum to right heart contractility. The severity of septal deviation and RV shape may predict the development of RV failure during LVAD support [5].

Further, the significance of TV insufficiency has been debated lately as an important factor for RV dysfunction in LVAD patients. [2] A preoperative evaluation of RV function and TV regurgitation may help early identification of patients at high risk for RV failure following LVAD placement.

## Methods

This study is a retrospective review of our patients’ data (n = 51) who underwent implantation of non-pulsatile LVAD from June 2006 to August 2010. The aim of this study is to evaluate the impact of preoperative RV dysfunction, TV competency, and septal contribution to RV function following implantation of an LVAD, as well as outcome and adverse events requiring readmission of patients to the hospital. The available echocardiography data were used to evaluated the RV function, septal deviation, and TV regurgitation while on LVAD support. The student *t* test was used to analyze the data. A p value of < 0.05 was considered as significant. This study was approved by the Institutional Review Board at the Montefiore Medical Center, Albert-Einstein College of Medicine.

## Results

**Figure 1:**
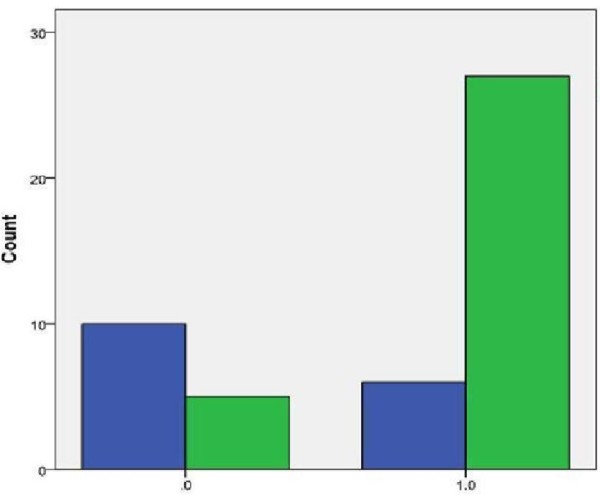
**Correlation of preoperative RV dysfunction vs postoperative RV failure.** A preoperative RV dysfunction predicated the postoperative RV failure following LVAD implantation (*p* = 0.002).

The mean age was 55.1 ± 13 years, with a male to female ratio of 3.25. The etiology included nonischemic dilatative cardiomyopathy (n = 17), ischemic cardiomyopathy (n = 27), postcardiac surgical shock (PCCS, n = 4), valvular (n = 1), and post partum cardiomyopathy (n = 2). The implanted devices included HeartMate II (n = 43), Ventriassist (n = 4), and HeartWare (n = 4). Additional procedures included tricuspid valve repair (n = 8), closure of PFO (n = 5), and aortic valve replacement (n = 5). The indication for assist device implantation included destination therapy (DT, n = 17), bridge to transplantation (BTT, n = 17), and potential bridge to transplantation (PBT, n = 17). The mean preoperative systolic pulmonary artery pressure (PAP) was 51.8±16.6 mmHg, and the pulmonary vascular resistance (PVR) was 3.58±1.83 woods. Preoperative cardiac index (CI) was 2.1±0.6, left ventricular ejection fraction (LVEF) was 21.97%±7.56%, with a median of 20%, and the preoperative RV work index was 564±372. Eighteen of 51 patients had intra-aortic balloon pump (IABP) in place. The intermacs level was 2.8±3.3, and the mean time of support was 314.5±235 with a median of 240 days at the time of closing this study. The preoperative RV dysfunction was diagnosed echocardiographically in 33 patients (65%), which correlated with postoperative worsening RV dysfunction in 32 patients (*p* = 0.001)(Figure 1). The preoperative TV regurgitation was seen in 31 of 37 patients in whom the TV was evaluated echocardiographically in preoperative period. The correlation between TV regurgitation and postoperative RV dysfunction was statistically not significant, which may be due to small sample size (*p* = 0.2). Septal position was evaluated in 49 patients, while a deviation towards the left ventricle was seen in 17 patients (34%), which correlated with RV dysfunction (*p* = 0.002) indicating the significant role of the septum in RV function. The 30-day mortality was 13.7% (7/51 patients), and the overall mortality since 6/13/2006 until 1/11/2010 was 23.5% (12/51 patients). Twenty-one patients (21/51; 41.2%) have undergone orthotopic heart transplantation successfully. The initial post-implant length of stay (LOS) was 34.8±22.9 days, and LOS in CSICU was 14.7±16.2 days (median 9 days). Twenty-six patients (51%) were discharged home and 25 patients (49%) to a rehabilitation facility. The average days spent out off hospital until the next admission was 86.7±139 and 90.9±154 day for patients who were discharged home or rehab (Table 1), respectively (the difference is statistically not significant).

**Table 1:** Postoperative complications on LVAD patients requiring readmission to Hospital

**Complication**	**Total number of readmissions**
GIB	22
GI discomfort, nausea, vomiting	6
Fever	7
Arrhythmias	3
AICD shock	5
CVA	2
TIA/seizures	4
Syncope	5
Driveline infection	8
Device malfunction	5
LVAD explant	2
Fluid overload	4
Respiratory failure	2
*Other	11

*Others: reduced LVAD flow 2, pneumonia 1, headaches 1, mucositis 1, pancytopenia 1, bactremia 1, change in mental status 1, PIC line infection 1, DKA 1.

## Discussion

Dynamic improvement in RV function is associated with favorable long-term outcome in patients with CHF [1]. Our study underscores the significance of preoperative RV dysfunction on postoperative worsening of RV dysfunction implantation of an LVAD (Figure 1*p* = 0.002). Optimization of right heart function prior to implantation of an LVAD may reduce the risk of RV failure post-LVAD placement. In cases of severe right heart failure, simultaneous temporary mechanical support of the right heart may be indicated. Drakos et al. [6]. reported the preoperative risk factors for the development of RV failure after LVAD implantation in a series of 175 patients, which included a preoperative need for intra-aortic balloon counterpulsation, increased pulmonary vascular resistance, and destination therapy. We believe that a scoring system would be beneficial in predicting RV dysfunction, and the need for temporary RV mechanical support or permanent biventricular support [7], however, such a scoring system would require a standardized prospective study. A focused preoperative and postoperative evaluation of hemodynamic and echocardiographic data of right heart function can be used to create a scoring system to predict the RV dysfunction during the LVAD support.

During the LVAD support, the negative pressure in LV created by the LVAD causes deviation of interventricular septum towards the LV, which eliminates the septal contribution to right heart contractility. The severity of septal deviation may help in adjusting the LVD flow under echocardiography guidance and reduce the septal deviation towards the LV, which may then limit the RV dysfunction while on LVAD support. The RV function will eventually improve in days to weeks, postoperatively [8], however, in the acute stage, prevention of acute RV failure and its management is crucial for LVAD patients’ survival [8,9]. Hendry et al. [10]. evaluated the role of septal shifting on RV dysfunction during the LVAD support. Septal shifting was documented as a change in LV shape index that was calculated using echocardiography findings. RV cardiac output decreased during LVAD support due to septal shifting towards the left ventricle, however, RV output was worse with increased RV afterload. This condition is tolerated by a normal functioning RV, however, in the presence of preoperative RV dysfunction and pulmonary hypertension, septal function is crucial for RV function, [10] and a deviation of the septum towards the LV will result in acute RV failure. Our study demonstrated that the patients with RV dysfunction have septal deviation towards the left ventricle (*p* = 0.02). The degree of septal shifting may predict the development of RV failure during LVAD support. This finding may help to adjust the flow following LVD placement and correct the septal deviation, which may then improve the RV dysfunction. A gradual increase of pump speed under echocardiography guidance can demonstrate the septal deviation and RV dysfunction immediately. An Adjustment of speed can be made under echocardiographic monitoring.

The importance of a competent TV on RV function has been underestimated. Potapov et al. 11 reported in a series of 54 patients that TV incompetence, RV dysfunction and shape, as well as right atrial dilation (short/long axis ratio >0.6), may help to predict the RV failure prior to LVAD placement [11]. In our series, the pre-existing TV regurgitation was associated with worsening RV function following LVAD implantation. However, this observation was not statistically significant (*p* = 0.29), which may be due to the small number of patients who had adequate echocardiographic evaluation of TV. Puwanant et al. [2]. emphasized the role of TVR in post-LVAD dysfunction of the RV. Lam et al. [4]. reported a small series of 21 patients that the RV function following LVAD implantation was better in the presence of a competent TV and was associated with an improved clinical condition of patients while on LVAD [4]. Preoperative evaluation of TV, RV function, shape, and geometry [11] may help to select patients who would benefit from biventricular support. In the presence of a significant TVR, we recommend a ring repair of the TV, which can be performed at the time of LVAD implantation.

In this study we also evaluated the pattern of adverse events causing readmission of LVAD patients to the hospital (Table 1). The adverse events in our patients are similar to reported data in the literature [12-18]. High risk of gastrointestinal bleeding (GIB) in LVAD patients has been reported in up to 40% of patients [13,18]. We had increased risk of GIB in our patients, however we didn’t identify any specific risk factor. The work up for GIB in LVAD patients is the same as in other patients. The anticoagulation can be reversed until the bleeding source is identified and controlled. Hayes et al. [18]. suggested using intravenous octreotide in LVAD patients who suffered GIB. Knowledge of the most frequent adverse events, which may mandate readmission to the hospital in a timely fashion, may prevent serious complications.

### Limitations of the study

This study has the common bias adherent to any retrospective study. Further, the study population was not uniform, and the etiology of heart failure was different. Some of the LVAD implantations were done up to 5 years ago and considering the improved techniques and advances in critical care, we should have fewer adverse events in coming years. The interobserver bias of clinicians who reviewed the echocardiograms is another limitation.

## Conclusion

An LVAD is an effective approach in bridging patients with end-stage heart failure to transplantation. Preoperative RV dysfunction is a predictor of post-implantation RV failure; however, the RV function may improve over time following LVAD implantation. In the presence of severe RV dysfunction, a temporary RVAD should be considered at the time of LVAD placement. An adequate medical adjustment of pulmonary hypertension and RV function In preoperative period may reduce the risk of RV failure during LVAD support. We recommend a TV ring repair at the time of LVAD implantation if there is echocardiographic evidence of significant TVR. An echocardiographic evaluation of RV function and septal deviation should be performed in patients following LVAD placement in the immediate postoperative period. An adjustment of LVAD flow based on echocardiographally monitored septal deviation can potentially prevent acute RV failure following LVAD implantation.

## Competing interests

The authors declare that they have no competing interests.

## Authors’ contributions

S N-M, D D, R B, R M, D D’A were actively involved in operative procedures and contributed equally in preparing the manuscript. The statistical analysis was performed by S N-M and R B. All authors read and approved the final manuscript.
